# Discoloration Improvement by Mechanically-Milled Binary Oxides as Radiopacifier for Mineral Trioxide Aggregates

**DOI:** 10.3390/ma15227934

**Published:** 2022-11-10

**Authors:** Hsiu-Na Lin, Ling-Chi Wang, May-Show Chen, Pei-Jung Chang, Pin-Yu Lin, Alex Fang, Chin-Yi Chen, Pee-Yew Lee, Chung-Kwei Lin

**Affiliations:** 1 Research Center of Digital Oral Science and Technology, College of Oral Medicine, Taipei Medical University, Taipei 110, Taiwan; 2 Department of Dentistry, Chang Gung Memorial Hospital, Taipei 105, Taiwan; 3 School of Dentistry, College of Oral Medicine, Taipei Medical University, Taipei 110, Taiwan; 4 Center of Dental Technology, Chang Gung Memorial Hospital, Linkou, Taoyuan 333, Taiwan; 5 Division of Prosthodontics, Department of Dentistry, Taipei Medical University Hospital, Taipei 110, Taiwan; 6 Graduate Institute of Manufacturing Technology, National Taipei University of Technology, Taipei 106, Taiwan; 7 Department of Optoelectronics and Materials Technology, National Taiwan Ocean University, Keelung 202, Taiwan; 8 Department of Engineering Technology and Industrial Distribution, Texas A & M University, College Station, TX 77843, USA; 9 Department of Materials Science and Engineering, Feng Chia University, Taichung 407, Taiwan; 10 School of Dental Technology, College of Oral Medicine, Taipei Medical University, Taipei 110, Taiwan

**Keywords:** mineral trioxide aggregates, radiopacifier, zirconia, hafnia, tantalum pentoxide, binary oxide, mechanical milling, radiopacity, discoloration

## Abstract

Mineral trioxide aggregates (MTA) have been widely used in endodontic treatments, but after some time, patients suffer tooth discoloration due to the use of bismuth oxide (Bi_2_O_3_) as a radiopacifier. Replacement of Bi_2_O_3_ with high energy ball-milled single (zirconia ZrO_2_; hafnia, HfO_2_; or tantalum pentoxide, Ta_2_O_5_) or binary oxide powder was attempted, and corresponding discoloration improvement was investigated in the present study. Bi_2_O_3_-free MTA is expected to exhibit superior discoloration. The radiopacity, diametral tensile strength, and discoloration of MTA-like cements prepared from the as-milled powder were investigated. Experimental results showed that MTA-like cements prepared using Ta_2_O_5_ exhibited a slightly higher radiopacity than that of HfO_2_ but had a much higher radiopacity than ZrO_2_. Milling treatment (30 min to 3 h) did not affect the radiopacities significantly. These MTA-like cements exhibited superior color stability (all measured ΔE_00_ < 1.0) without any perceptible differences after UV irradiation. MTA-like cements prepared using ZrO_2_ exhibited the best color stability but the lowest radiopacity, which can be improved by introducing binary oxide. Among the investigated samples, MTA-like cement using (ZrO_2_)_50_(Ta_2_O_5_)_50_ exhibited excellent color stability and the best overall performance with a radiopacity of 3.25 mmAl and a diametral tensile strength of 4.39 MPa.

## 1. Introduction

When dental pulp is exposed to bacteria, endodontic treatment is required to repair and save the infected tooth. Endodontic treatment involves a series of steps that include the removal of diseased pulp, clearance of root canal infection, medication if necessary, and sealing the root canal. Presently, mineral trioxide aggregates (MTA) possessing superior obturation ability and biocompatibility have been widely used in the repair of lateral perforations, apexification, direct pulp capping, and root end filling [1,2,3].

Typically, MTA is a powder mixture consisting of 75% Portland cement, 20% radiopacifier, and 5% gypsum. Though widely used in endodontic treatment as dental filling material, MTA still experiences a few drawbacks, including a long setting time, tooth discoloration, difficulty in handling, and relatively high cost [4,5,6]. Long setting time is the first inevitable incident to be encountered. Numerous investigations have been performed to reduce the initial and final setting time. For instance, not only calcium-silicate-based cements [7,8,9] but also various solidifying solutions [10,11] can lessen the setting time issue. Tooth discoloration, however, will become a major concern after clinical treatment, especially under the consideration of aesthetic dentistry.

Blood contamination and constitution within MTA are the major causes of tooth discoloration. It has been reported that hemoglobin and hematin molecules within the blood will deepen the tooth color after clinical therapy [5,12], whereas various metal oxides within the original and commercially available MTA (i.e., ProRoot®), including Fe_2_O_3_, Al_2_O_3_, MgO, and especially the radiopacifier Bi_2_O_3_ are responsible for tooth staining. Thus, Biodentine® and BioAggregate® use zirconium oxide (ZrO_2_) and tantalum pentoxide (Ta_2_O_5_), respectively, as alternative radiopacifiers to resolve tooth discoloration [13,14]. Suitable radiopacifiers to improve the performance of MTA remains an interest of research and development. For instance, previous investigations using either a physically high-energy ball milling process [15,16] or chemically wet methods such as precipitation, sol-gel, and spray pyrolysis processes [17,18,19] to modify bismuth oxide by introducing a secondary oxide have been attempted, and improvements in radiopacity performance were observed. These MTA-like cements, however, may experience tooth discoloration after clinical service due to the presence of bismuth.

Among various techniques to prepare radiopacifier for MTA, the high-energy ball milling process [20,21,22] is a unique method for synthesizing materials that are difficult to prepare using conventional techniques. By continuous milling of a single starting powder or alloying of multiple components, amorphous materials, extended solid solutions, intermetallic compounds, metastable crystalline materials, nanocrystalline powders, and quasicrystals can be synthesized [20,22,23,24]. For instance, the high-temperature metastable δ-Bi_7.38_Zr_0.62_O_2.31_ phase was synthesized by mechanically milling a Bi_2_O_3_ and ZrO_2_ powder mixture [15], whereas the formation of β-Bi_7.8_Ta_0.2_O_12.2_, and δ-Bi_3_TaO_7_ phases can be achieved by milling binary Bi_2_O_3_ and Ta_2_O_5_ [16]. Corresponding radiopacities of various as-milled Bi_2_O_3_–based composite powders have been examined [15,16]. The effect of milling time, oxide addition, and storage environment on the radiopacity performance have been discussed. The long-term clinical issue of tooth discoloration due to the presence of Bi_2_O_3_, however, has not been addressed. Since Bi_2_O_3_-free MTA can improve tooth discoloration [13,14], in the current work, single and binary oxide powder will be treated with a high-energy ball milling process, and the as-milled powder will serve as the radiopacifier for MTA. The radiopacity performance and tooth discoloration of these MTA-like cements will be investigated to reveal their potential for clinical application.

## 2. Materials and Methods

### 2.1. Preparation and Characterization of Mechanically-Milled Radiopacifier

A shaker ball mill (SPEX 8000D, Fisher Scientific, Ottawa, ON, Canada) was used to perform the ball milling process [25]. It was operated in an Ar-filled glove box (the total oxygen and water concentration was less than 100 ppm). Bismuth, zirconium, hafnium, and tantalum oxide powder were used as the starting powders. A total weight of 6 g of either single (all four oxides) or desired binary oxide mixture (A_100_B_100-x_ in weight percentage; A and B are ZrO_2_, HfO_2_, and Ta_2_O_5_) was canned into an SKH 9 high-speed steel vial (40 mm and 50 mm in diameter and height, respectively) together with 7 mm Cr-steel balls (~30 g). To investigate the effect of powder refinement, the overall processing time was set at 3 h and interrupted every 30 min to cool down the vial. An X-ray diffractometer (Bruker AXS GmbH-D2 PHASER, Billerica, MA, USA) was used to inspect the crystalline structure of the as-milled powders at various milling stages using Cu Kα emission filtered by nickel. The resulting grain size was estimated with Scherrer’s formula using a shape factor (k) of 0.9 [26].

### 2.2. Preparation and Characterization of MTA-Like Cements

The as-milled powders were used as the radiopacifier for mineral trioxide aggregates (MTA). MTA-like cements were prepared by mixing Portland cement/radiopacifier/calcium sulfate (75/20/5 in wt.%) with a benchtop ball mill (Retsch PM100, Haan, Germany) for a duration of 10 min. Thereafter, the mixed powder was further mixed with deionized water (powder/water ratio equals 3) and placed into a mold (10 mm diameter and 1 mm thickness). The samples were settled at 37 °C for another 24 h in an incubator with 100% relative humidity to simulate the oral environment. For each MTA-like cement, six disc samples were prepared and radiographed by a dental X-ray system (VX-65; Vatech Co, Yongin Si Gyeonggi-Do, Korea) using 62 kV voltage and 10 mA current density. A radiographic film (Koadak CR imaging plate size 2; Eastman-Kodak Co, Rochester, NY, USA) located at 30 cm apart and exposed for 0.64 s was used to take the radiographic image. Six disc samples and a referenced aluminum step-wedge were examined at the same time. The gray values of each step for the aluminum wedge and the samples were determined using Image J software (version 1.53s, Wayne Rasband, National Institutes of Health, Bethesda, MD, USA) to assess the radiopacity of the MTA-like cements. For diametral tensile strength (DTS) tests, the cements (*n* = 6) were loaded into a cylindrical acrylic mold (6 mm diameter × 5 mm height) and placed in an environment-controlled incubator (37 °C with 100% relative humidity) for 1 day. A universal testing machine (CY-6040A8, Chun Yen testing machines, Taichung, Taiwan) was used to perform the DTS test at a crosshead speed of 6.0 mm/min. The DTS value was calculated according to the following equation: DTS = 2F/πbw, where F is the maximum applied load (N), and b and w are the diameter (mm) and the height (mm) of the sample, respectively. Detailed experimental procedures are available elsewhere [16].

### 2.3. Discoloration Examination of MTA-Like Cements

The accelerated discoloration experiments were performed by immersing the MTA-like cements in 2 mL glycerin (Wako, Osaka, Japan) for 15 min [27]. The soaked specimens were then irradiated by a UV curing machine (Phrozen Cure V2, Hsinchu, Taiwan) that used UV-LEDs (365 nm, 385 nm, and 405 nm, 60 W in total) with 360° counterclockwise rotation at a speed of 3 rpm. The color of the specimens (*n* = 6) was measured by a digital dental colorimeter (OptiShade Styleitaliano, St-Imier, Switzerland) to obtain the L*a*b* values. △E_00_ of the treated specimens were computed by comparing them to the specimens without UV irradiation according to the CIE (International Commission on Illumination) standard [28].

### 2.4. Statistical Analysis

For statistical analysis, the sample size was six for radiopacity, DTS, and discoloration of MTA-like cements. Student’s paired *t*-test was used to perform the statistical analysis using SPSS software (version 18.0, IBM Corporation, NY, USA). The radiopacities, DTS, and △E_00_ between paired samples were compared with various confidence intervals ranging from 0.01 to 0.001.

## 3. Results and Discussion

### 3.1. Mechanically Milled Single Oxide as Radiopacifier

**Figure 1:**
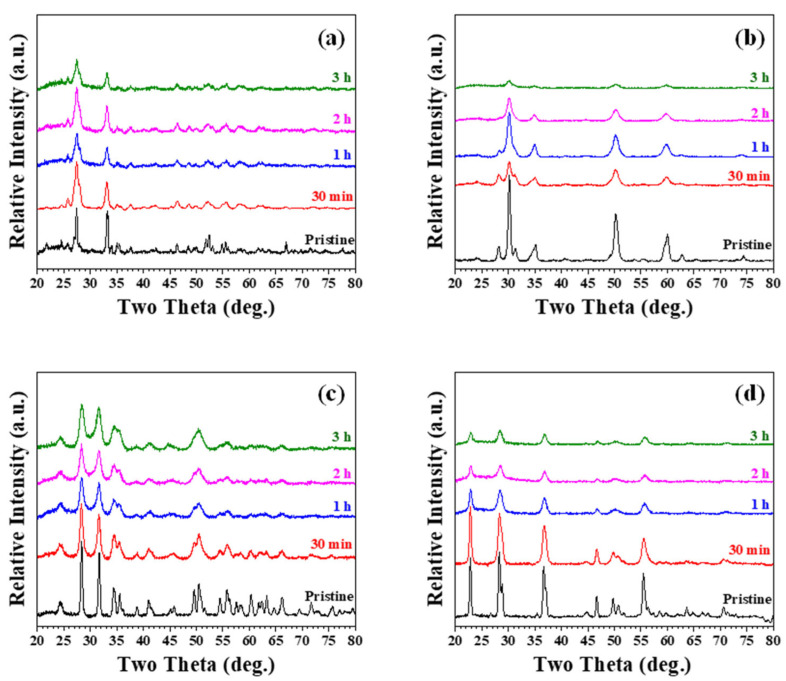
X-ray diffraction patterns of high energy ball-milled (**a**) Bi_2_O_3_, (**b**) ZrO_2_, (**c**) HfO_2_, and (**d**) Ta_2_O_5_ after different milling times.

Figure 1 shows the X-ray diffraction patterns of mechanically milled oxides (Bi_2_O_3_, ZrO_2_, HfO_2_, and Ta_2_O_5_) after different milling times. Since all these oxides are brittle, continuous refinement of starting oxide powders due to the high-energy ball milling process can be observed. Figure 1a shows the XRD patterns of mechanically milled α-Bi_2_O_3_ (pristine starting powder, PDF No. 27-0053). Peaks’ intensities decreased and the peaks broadened with increasing milling time. It is, however, interesting to note that mechanically milled ZrO_2_ exhibited slightly different behavior compared with the continuous refinement of Bi_2_O_3_ shown in Figure 1a. As shown in Figure 1b, after short milling treatment (before 1 h of milling), the starting tetragonal phase ZrO_2_ (PDF No. 50-1089) gradually transformed into cubic phase ZrO_2_ (PDF No. 49-1642). After 2 h of milling, tetragonal-to-cubic phase transformation was completed, and a single cubic phase ZrO_2_ was exhibited. Further milling induced continuous refinement of ZrO_2_ particles similar to that of α-Bi_2_O_3_. Figure 1c,d shows similar behavior as that of α-Bi_2_O_3_ (Figure 1a), for which continuous refinement of starting powders (monoclinic HfO_2_, PDF No. 34-0104; orthorhombic Ta_2_O_5_, PDF No. 25-0922) resulted in peak broadening.

**Figure 2:**
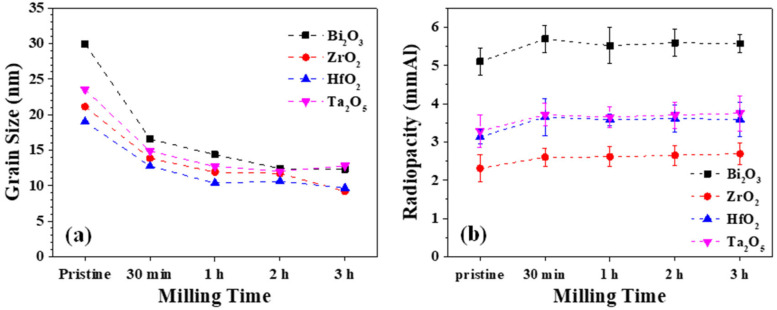
Variation of (**a**) grain size and (**b**) radiopacity for as-milled oxide powder as a function of milling time.

Continuous ball milling with high energy input can induce phase transformation of the metastable phase. It has been reported that ZrO_2_ can experience monoclinic-to-cubic transformation using a planetary ball mill. For instance, Gorodylova et al. [29] revealed that monoclinic-to-tetragonal phase transformation could be observed after 48 h of milling, whereas further transformation to cubic phase ZrO_2_ will require a long 150 h of milling. It can be significantly shortened to 2 h by using a high-energy SPEX ball mill as reported in the present work. Figure 2a shows the evolution in grain size estimated by Scherrer’s formula [26] of the corresponding as-milled oxide powder. It can be noted that a significant decrease in grain size (all *p*-value = 0.000) can be observed after 30 min of high-energy ball milling treatment. At the same time, no significant decrease (all *p*-value ≥ 0.100) can be noticed thereafter. After mixing them into MTA-like cements, the radiopacities of the as-milled oxide were measured, and Figure 2b shows the corresponding results. As a general trend, the radiopacities for those MTA-like cements prepared using pristine oxides were smaller than those of the as-milled oxides but significantly larger (all *p*-value = 0.000) than those without radiopacifier (Portland cement only). For bismuth oxide, the original radiopacifier used in MTA, the radiopacity was 5.10 mmAl before milling and increased to 5.69, 5.52, 5.59, and 5.57 mmAl, after 30 min, 1 h, 2 h, and 3 h of milling, respectively. The radiopacity was 2.31, 3.13, and 3.28 mmAl for pristine ZrO_2_, HfO_2_, and Ta_2_O_5_, respectively. It increased to 2.65, 3.65, and 3.71 mmAl for 30 min as-milled oxide powder, respectively. However, there are no unambiguous differences in radiopacities that can be observed for those after different milling times. The atomic number of the radiopacifier within the MTA basically affects its radiopacity. The atomic numbers are 83, 40, 72, and 73 for Bi, Zr, Hf, and Ta elements, respectively. The measured radiopacity follows this trend. The increase in radiopacity after milling treatment is probably due to the particle size refinement. Prolonged milling, however, did not improve the radiopacity further. This shows a similar result as reported in the literature. Table 1 summarizes all the radiopacities prepared using the single oxides investigated in the present study.

**Figure 3:**
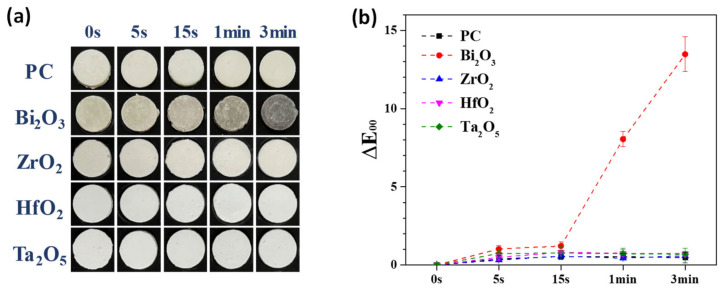
(**a**) Photos and (**b**) corresponding ΔE_00_ values for MTA-like cements prepared using as-milled oxide powder and irradiated under UV exposure.

Since the 30 min as-milled powder exhibited relatively large radiopacity and short milling process time, it was used as the radiopacifier to prepare MTA-like cements for the accelerated discoloration experiments [27]. Figure 3a shows the corresponding images of MTA-like cements immersed in glycerol and UV exposure for different times. Among these samples, t he MTA-like cements prepared using bismuth oxide exhibited obvious discoloration after UV exposure, whereas the other samples revealed no visible differences after UV exposure. In order to further examine the differences, CIE L*a*b* values of these samples were measured, and Figure 3b shows the corresponding results. For Portland cement without a radiopacifier, the values for ΔE_00_ were 0.38, 0.53, 0.53, and 0.46 after UV exposure for 5 s, 15 s, 1 min, and 3 min, respectively. Bi_2_O_3_-added MTA-like cements exhibited a significant difference. It can be noted that after relatively short UV exposure (say 15 s.) ΔE_00_ was 1.20 (*p*-value = 0.138) and reached 8.05 after 1 min. (*p*-value = 0.000), and further increased to 13.48 at the end of the accelerated discoloration experiment (3 min) (*p*-value = 0.000). It should be pointed out that after 1 min of UV exposure, the value of ΔE_00_ already exceeded the perceptible clinical level of 3.7 [30]. ZrO_2_, however, possessed more stable color stability after UV exposure. ΔE_00_ was 0.32, 0.56, 0.44, and 0.58 as a function of UV exposure time, respectively. HfO_2_ and Ta_2_O_5_ exhibited similar ΔE_00_ results and were 0.71 and 0.68, respectively, at the end of exposure. Table 2 summarizes all the ΔE_00_ results for discoloration experiments.

### 3.2. Mechanically Milled Binary Oxides as Radiopacifier

**Figure 4:**
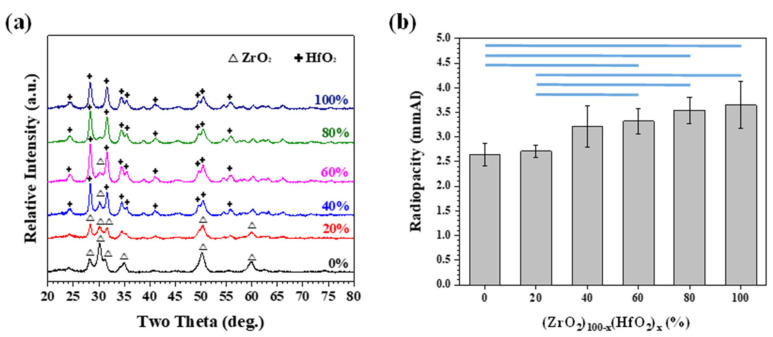
(**a**) X-ray diffraction patterns of as-milled (ZrO_2_)_100-x_(HfO_2_)_x_ powder and (**b**) corresponding radiopacities of as-prepared MTA-like cements. The horizontal blue lines indicated that the paired results were statistically different at a confidence level of 95%.

In the previous section, we demonstrated that MTA-like cements with alternative oxide (ZrO_2_, HfO_2_, and Ta_2_O_5_) radiopacifier can significantly lessen discoloration. The radiopacity, however, may not be able to meet the ISO 6876:2012 requirement (>3 mmAl). Using binary oxide as a radiopacifier for MTA has been attempted. Figure 4 shows the XRD patterns and their corresponding radiopacities for (ZrO_2_)_100-x_(HfO_2_)_x_ (x = 0, 20, 40, 60, 80, and 100) powder mixture after 30 min of high-energy ball milling treatment. As shown in Figure 4a, it can be noted that the XRD patterns of the as-milled powers exhibited the diffraction peaks of the pristine powders (i.e., ZrO_2_ and HfO_2_), and no distinct metastable phase formation can be noticed. The radiopacities shown in Figure 4b basically follow the law of mixture. The radiopacity was 2.65 and 3.65 mmAl for pristine ZrO_2_ and HfO_2_. The radiopacity for binary ZrO_2_ and HfO_2_ powder gradually increased with an increasing amount of HfO_2_ addition. With 40% of HfO_2_, the radiopacity was 3.22 mmAl and met the ISO standard requirement. Statistical analysis was performed to further reveal the differences in radiopacities by comparing different sets of samples where the radiopacity was statistically different at a confidence level of 95% (marked as horizontal blue lines in Figure 4b) for HfO_2_-rich (HfO_2_ ≥ 60%) and ZrO_2_-rich (ZrO_2_ ≥ 80%) MTA-like cements.

**Figure 5:**
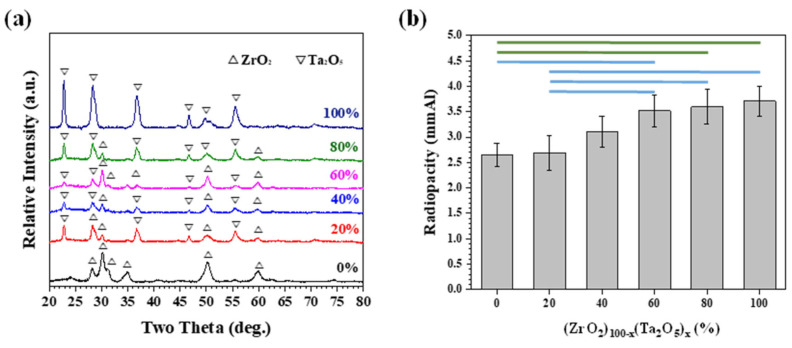
(**a**) X-ray diffraction patterns of as-milled (ZrO_2_)_100-x_(Ta_2_O_5_)_x_ powder and (**b**) corresponding radiopacities of as-prepared MTA-like cements. The horizontal green and blue lines indicated that the paired results are statistically different at a confidence level of 99 and 95%, respectively.

**Figure 6:**
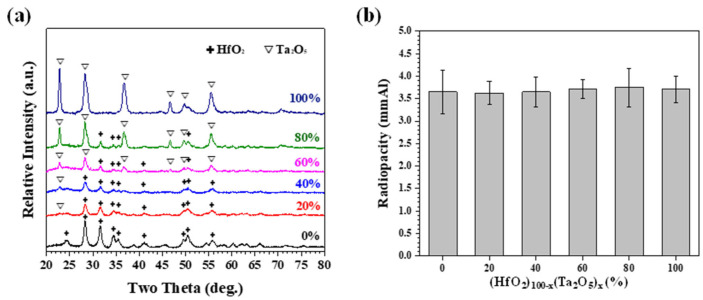
(**a**) X-ray diffraction patterns of as-milled (HfO_2_)_100-x_(Ta_2_O_5_)_x_ powder and (**b**) corresponding radiopacities of as-prepared MTA-like cements.

A similar trend can be observed for (ZrO_2_)_100-x_(Ta_2_O_5_)_x_ (x = 0, 20, 40, 60, 80, and 100) powder. The XRD patterns possessed a combination of the refined pristine ZrO_2_ and Ta_2_O_5_ diffraction peaks due to high-energy ball milling (Figure 5a). In contrast, the radiopacities increased as a function of Ta_2_O_5_ addition (Figure 5b) and were higher than 3 mmAl with 40 wt.% Ta_2_O_5_ of addition. This can be attributed to the similar radiopacity performance between HfO_2_ (3.65 mmAl) and Ta_2_O_5_ (3.71 mmAl). The radiopacities for Ta_2_O_5_ -rich (Ta_2_O_5_ ≥ 60%) and ZrO_2_-rich (ZrO_2_ ≥ 80%) MTA-like cements were statistically different at a confidence level of 95% or 99%, marked as horizontal blue and green lines in Figure 5b, respectively). The slightly larger radiopacity of Ta_2_O_5_ than that of HfO_2_, however, slightly increased the statistical difference to a confidence level of 99% for two sets of data. Figure 6 shows the results of (HfO_2_)_100-x_(Ta_2_O_5_)_x_ (x = 0, 20, 40, 60, 80, and 100) powder where XRD patterns exhibited both HfO_2_ and Ta_2_O_5_ diffraction peaks (Figure 6a) and no significant differences in radiopacities can be observed (Figure 6b).

### 3.3. Performance of Equivalent Binary Oxide Radiopacifier

**Figure 7:**
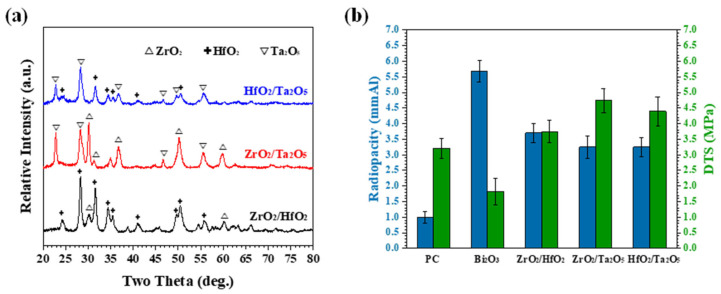
(**a**) X-ray diffraction patterns of as-milled binary oxide powder and (**b**) the radiopacities and DTS and MTA-like cements prepared by binary oxide powder. PC and Bi_2_O_3_-MTA are shown for comparison.

As shown in the previous section, it can be noted that the radiopacities of the MTA-like cements prepared using binary oxide basically followed the law of mixture. In order to meet the ISO requirement (3 mmAl), the addition of low radiopacity ZrO_2_ (2.65 mmAl) must be less than 40 wt.%. Therefore, the equivalent binary oxide was used as the prototype to investigate further its feasibility as a radiopacifier in MTA for clinical application. Figure 7a shows the XRD patterns of equivalent binary oxide powders that followed the same trend as addressed in Figure 4a, Figure 5a, and Figure 6a, respectively. The radiopacities, Figure 7b, were 3.25, 3.25, and 3.70 mmAl for (ZrO_2_)_50_(HfO_2_)_50_, (ZrO_2_)_50_(Ta_2_O_5_)_50_, and (HfO_2_)_50_(Ta_2_O_5_)_50_, respectively. All were higher than 3 mmAl, significantly better (*p*-value = 0.000) than its radiopacifier-less counterpart (Portland cement, 1.00 mAl) and lower than the Bi_2_O_3_-added MTA-like cement (5.69 mmAl). In contrast, the corresponding DTS (also shown in Figure 7b) was 4.74, 4.39, and 3.75 MPa, higher than Portland cement (3.21 MPa) and Bi_2_O_3_ (1.82 MPa).

**Figure 8:**
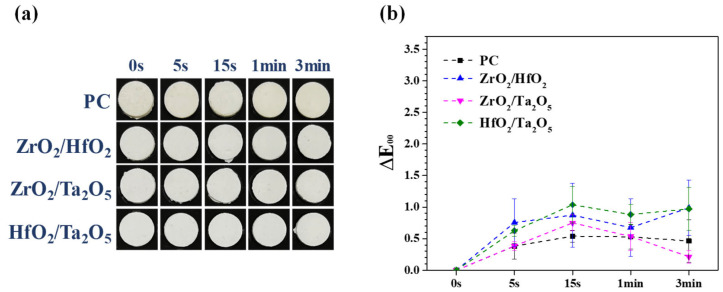
(**a**) Photos and (**b**) corresponding ΔE_00_ values for MTA-like cements prepared using as-milled binary oxide powder and irradiated under UV exposure.

Figure 8 shows the practical images after accelerated discoloration experiments for the MTA-like cements prepared using equivalent binary oxides. It can be noted that all the samples exhibited excellent color stability and no perceptible difference. Recalling the results from using single oxide of ZrO_2_, HfO_2_, and Ta_2_O_5_, the values of ΔE_00_ were 0.58 ± 0.29, 0.71 ± 0.15, and 0.68 ± 0.39, respectively. The ΔE_00_ values were 0.99 ± 0.43, 0.21 ± 0.10, and 0.97 ± 0.34 for (ZrO_2_)_50_(HfO_2_)_50_, (ZrO_2_)_50_(Ta_2_O_5_)_50_, and (HfO_2_)_50_(Ta_2_O_5_)_50_, respectively. MTA-like cement prepared using equivalent (ZrO_2_)_50_(Ta_2_O_5_)_50_ binary oxide exhibited excellent color stability during simulated UV exposure experiments.

It is well known that MTA with Bi_2_O_3_ as a radiopacifier can induce immediate or delayed tooth discoloration. As reported in the literature, alternative radiopacifiers include ZrO_2_ (ENDOCEM Zr, RetroMTA, and Biodentine) and Ta_2_O_5_ (NeoMTA Plus and BioAggregate), can lessen tooth discoloration [13,14,27,31,32]. In the present work, not only ZrO_2_ and Ta_2_O_5_ but also HfO_2_ show superior color stability (all ΔE_00_ values were less than 1.00). This can be improved further by using (ZrO_2_)_50_(Ta_2_O_5_)_50_ as a radiopacifier within the MTA-like cement. The ΔE_00_ value was only 0.21 ± 0.10 (the lowest in the current investigation) and implied its excellent color stability.

## 4. Conclusions

In the present study, the single or binary oxide was treated using a high-energy ball milling process, which resulted in continuous refinement of starting powders. The radiopacity of as-milled powder after various periods of milling (30 min to 3 h), however, exhibited no significant differences, whereas the MTA-like cements prepared using either single or binary oxide exhibited superior color stability where all the measured ΔE_00_ values were less than 1.0 and no perceptible differences can be revealed. Among the investigated single oxides, ZrO_2_ was the best, with a ΔE_00_ value of 0.58. Its relatively low radiopacity (2.65 mmAl), however, may restrain its practical application. MTA-like cements prepared using equivalent (ZrO_2_)_50_(Ta_2_O_5_)_50_ binary oxide exhibited a radiopacity of 3.25 ± 0.30 mmAl, a DTS of 4.39 MPa, and excellent color stability (ΔE_00_ = 0.21). They will be investigated further for their feasibility in clinical applications.

## Figures and Tables

**Table 1:** Radiopacities of cements prepared using oxide radiopacifier after ball milling treatment.

	Milling Time	Pristine	30 min	1 h	2 h	3 h
Material						
Portland cement		0.91 ± 0.14	1.00 ± 0.18	N.A.	N.A.	N.A.
Bi_2_O_3_		5.10 ± 0.36	5.69 ± 0.35	5.52 ± 0.48	5.59 ± 0.35	5.57 ± 0.24
ZrO_2_		2.31 ± 0.36	2.65 ± 0.23	2.61 ± 0.26	2.65 ± 0.26	2.69 ± 0.29
HfO_2_		3.13 ± 0.19	3.65 ± 0.48	3.58 ± 0.16	3.61 ± 0.36	3.59 ± 0.44
Ta_2_O_5_		3.28 ± 0.42	3.71 ± 0.30	3.65 ± 0.27	3.70 ± 0.34	3.75 ± 0.46

**Table 2:** ΔE_00_ results for the accelerated discoloration experiments.

	UV Exposure	0 s	5 s	15 s	1 min	3 min
Material						
Portland cement		0.00	0.38 ± 0.21	0.54 ± 0.09	0.53 ± 0.20	0.46 ± 0.33
Bi_2_O_3_		0.00	1.02 ± 0.22	1.21 ± 0.26	8.05 ± 0.48	13.48 ± 1.11
ZrO_2_		0.00	0.32 ± 0.08	0.56 ± 0.09	0.44 ± 0.12	0.58 ± 0.29
HfO_2_		0.00	0.49 ± 0.18	0.81 ± 0.01	0.75 ± 0.22	0.71 ± 0.15
Ta_2_O_5_		0.00	0.73 ± 0.25	0.76 ± 0.41	0.72 ± 0.36	0.68 ± 0.39

## Data Availability

Not applicable.
